# Identification and characterization of LEA gene family in physic nut and functional analysis of *JcLEA1* under drought stress

**DOI:** 10.3389/fpls.2026.1759018

**Published:** 2026-01-30

**Authors:** Yuehui Tang, Xiaohui Wang, Shujing Wang, Xuechun Li, Xinxin Bao, Siqiong Xu, Dafei Liu, Wenxia Zhang, Chenyu Jiao

**Affiliations:** 1 College of Life Science and Agronomy, Zhoukou Normal University, Zhoukou, Henan, China; 2 Field Observation and Research Station of Green Agriculture in Dancheng County, Zhoukou, Henan, China; 3 School of Journalism and Communication, Zhoukou Normal University, Zhoukou, Henan, China

**Keywords:** abiotic stress, drought tolerance, *JcLEA1*, LEA gene family, physic nut

## Abstract

**Introduction:**

Late Embryogenesis Abundant (LEA) proteins are highly hydrophilic, glycine-rich proteins that accumulate during late seed ripening and play critical roles in abiotic stress responses. However, only a limited number of LEA genes have been functionally characterized in the drought-tolerant species physic nut, and systematic investigations of their characteristics and transcriptional dynamics remain unexplored.

**Methods and results:**

In this study, we identified 24 *LEA* genes (*JcLEAs*) in physic nut, which were systematically categorized into eight evolutionary subgroups (LEA*1* to *LEA*6, DHN, SMP) through comparative phylogenetic clustering with homologs from rice and Arabidopsis. Among the 24 *JcLEA* genes, most were predominantly expressed in seeds, with notably elevated transcript levels during the late seed maturation stage. RNA-seq data revealed that 13 *JcLEA* genes were responsive to one or more abiotic stress conditions (drought or salinity) in root tissues at multiple time points. Subcellular localization experiments in Arabidopsis protoplasts confirmed nuclear localization of *JcLEA*1, and transgenic Arabidopsis plants overexpressing *JcLEA1* exhibited enhanced drought resilience compared to wild-type, as indicated by reduced relative electrolyte leakage and MDA content, elevated proline accumulation and betaine content, and enhanced superoxide dismutase activity under drought conditions. Further analysis of transgenic plants overexpressing *JcLEA1* subjected to drought stress confirmed the functional role of *JcLEA* genes in drought tolerance.

**Discussion:**

This study provides the first in-depth genomic characterization of the LEA gene family members in physic nut, complemented by functional investigations that advance our understanding of its role in abiotic stress adaptation. Our findings offer a foundation for molecular breeding strategies to improve drought tolerance in bioenergy crops, particularly physic nut.

## Introduction

Abiotic stress significantly reduces agricultural productivity worldwide. To counteract environmental challenges, plants have evolved sophisticated response systems capable of perceiving external stimuli and transducing these signals through specific biochemical pathways, ultimately triggering transcriptional reprogramming of stress-related genes. Transcription factors, such as MYB, ERF, HD-Zip, and LEA proteins, have been identified as critical regulators mediating these biological processes (Mohanty and Hembram, 2025; Ma et al., 2024; Wang et al., 2024a; Zhang et al., 2025).

LEA proteins, characterized by pronounced hydrophilicity and intrinsically disordered structure, are instrumental in safeguarding cellular structures under dehydration stress (Hundertmark and Hincha, 2008). During late stages of seed maturation, small hydrophilic LEA proteins, gradually accumulate. These proteins, rich in alanine, glycine, and serine, exhibit conserved sequence motifs characterized by repetitive hydrophilic amino acid arrangements. They are first identified in the cotyledons of cotton embryos during late developmental stages (Dure et al., 1981), and have since been detected in diverse plant species (Mohanty and Hembram, 2025). Based on conserved motifs analysis and amino acid sequence similarity, 85 LEA proteins composed of 34 rice OsLEA and 51 Arabidopsis AtLEA, are categorized into nine distinct groups: LEA_1, LEA_2, LEA_3, LEA_4, LEA_5, LEA_6, dehydrin (DHN), seed maturation protein (SMP), and AtM (Hundertmark and Hincha, 2008; Wang et al., 2007).

LEA proteins confer cellular protection through multiple mechanisms that are essential for plant survival under abiotic stress, including ion sequestration, macromolecular stabilization, and reactive oxygen species (ROS) scavenging. For example, *OsLEA1a* enhances plant resilience under abiotic stress conditions by reducing cell membrane degradation and enhancing ROS detoxification (Wang et al., 2021), while transgenic rice overexpressing *OsLEA4* exhibits markedly increased tolerance to drought, salinity, and heavy metal toxicity through improved cellular protection (Hu et al., 2016). In tobacco, *GiLEA5-2.1* improves drought and salt tolerance via direct interaction with catalase to scavenge ROS (Zhang et al., 2024). Similarly, *GhLEA-5* in cotton increases salt tolerance by promoting metabolic activity (Tian et al., 2025), and the *LEA4–4* gene enhances salt tolerance in Arabidopsis by strengthening cellular stress responses (Jia et al., 2020). Beyond stress adaptation, LEA proteins regulate plant growth and developmental processes. For instance, *TaLEA-1A* in wheat modulates seed quiescence and emergence by regulating the ABA (abscisic acid) and GA (gibberellic acid) balance (Lei et al., 2024), and LEA proteins contribute to kernel development and dehydration tolerance (Hundertmark and Hincha, 2008). Additionally, in Arabidopsis, dark-induced leaf senescence is mediated by the LEA protein ABR, which is negatively regulated by ABI5 (Su et al., 2016). In summary, although LEA protein functions have been widely investigated across diverse species, research on these proteins within the Euphorbiaceae family remains limited. This gap is particularly striking in physic nut, a drought-tolerant Euphorbiaceae species. Notably, drought tolerance in physic nut is mediated by a complex network involving LEA proteins alongside other genes, proteins, and metabolites, and our study focuses on characterizing the specific roles of LEA proteins within this network.

As a model species for drought adaptation, physic nut has gained prominence as a promising biodiesel feedstock owing to its remarkable environmental resilience and economic potential (Openshaw, 2000). This perennial shrub thrives in marginal environments characterized by poor soil fertility, water scarcity, and saline-alkaline stress, positioning it as a genetic model system for studying stress tolerance. The species’ most distinctive trait is its remarkably high seed kernel oil content (>60%), which serves as a renewable lipid source for biodiesel production (Openshaw, 2000). Critically, its ability to grow on non-arable land avoids direct competition with food crops, establishing it as a strategic candidate for bioenergy production and ecological restoration in degraded ecosystems. In this study, we performed a comprehensive genomic characterization of the LEA gene family in physic nut, including structural analysis, chromosomal localization, phylogenetic relationships, conserved motif identification, and *cis*-regulatory element analysis of promoter regions. We further analyzed expression patterns across various tissues, seed developmental stages, and abiotic stress conditions (salinity and drought). Finally, we validated *JcLEA1* function through heterologous expression in Arabidopsis and phenotypic characterization of transgenic lines. Our findings establish a critical foundation for elucidating LEA-mediated stress adaptation in physic nut and designing molecular breeding approaches to improve abiotic stress tolerance.

## Materials and methods

### Plant material

The inbred cultivar GZQX0401 of *Jatropha curcas* (*J. curcas*) was used in this study, as its genome has been fully sequenced (Wu et al., 2015). Its seeds were acquired from the South China Botanical Garden, Chinese Academy of Sciences, Guangzhou, China. The wild-type *Arabidopsis thaliana* (Arabidopsis) used was the Columbia ecotype (Col-0), with its seeds also obtained from the above-mentioned institution.

### Identification of LEA protein in physic nut

Using Arabidopsis (51 LEA proteins) and rice (34 LEA proteins) sequences as queries, a genome-wide BLASTP search was executed on the physic nut database to detect LEA family members. All candidates underwent Pfam (http://pfam.xfam.org/) and SMART (http://smart.embl-heidelberg.de/) database screening to confirm their classification (Letunic and Bork, 2018). The ExPASy ProtParam server (https://web.expasy.org/protparam/) was employed for systematic analysis of LEA protein physicochemical properties (Gasteiger et al., 2003).

### Phylogenetic analysis of LEA proteins

NCBI (https://www.ncbi.nlm.nih.gov/) provided the physic nut LEA protein dataset, whereas Arabidopsis and rice LEA sequences (51 from TAIR (https://www.arabidopsis.org/) ; 34 from NCBI) were integrated as reference sets. ClustalX-driven multiple alignments formed the basis for phylogenetic analysis. MEGA10.0 applied the Maximum Likelihood method with 1000 bootstrap replicates to establish evolutionary relationships. iTOL (https://itol.embl.de/) enabled high-resolution tree visualization.

### Exon-intron structure and conserved motif analysis

The GFF3 genome annotation file of physic nut provided gene structure data for LEA family members. Gene structure visualization (exon/intron organization) was conducted via the GSDS platform (http://gsds.gao-lab.org), followed by exon-intron composition assessment. Conserved motifs were identified using MEME (https://meme-suite.org/meme/tools/meme) with parameter settings: 20 motifs, width range 6–60 residues, and site distributions restricted to ≤1 occurrence per sequence. Final motif diagrams were annotated with TBtools (https://github.com/CJ-Chen/TBtools) (Chen et al., 2023).

### Chromosome localization analysis

The chromosomal locations of the LEA family genes in physic nut were determined using the NCBI online database (https://www.ncbi.nlm.nih.gov/). These positions were further confirmed by analyzing the physic nut genome annotation file (GFF3). TBtools software facilitated the construction of a chromosomal localization map for physic nut LEA genes (Chen et al., 2023).

### Analysis of cis-acting elements in *JcLEA* gene promoters

The 2000-bp promoter regions (upstream of ATG start codons) of *JcLEA* genes were acquired from NCBI. Cis-element prediction was executed on the PlantCARE platform (http://bioinformatics.psb.ugent.be/webtools/plantcare/html/), followed by TBtools-based visualization of element distribution patterns (Chen et al., 2023).

### LEA proteins interaction network analysis

The STRING database (https://string-db.org/) facilitated protein interaction prediction among physic nut LEA family members, followed by network topology construction in Cytoscape 3.10.0.

### Analysis of *JcLEAs* gene expression profile

Full-grain seeds of physic nut were selected and soaked in 0.5% potassium permanganate solution for 2 hours for sterilization. Subsequently, the seeds were germinated at 26°C in the dark for 5 days and directly sown in large round pots containing soil (30%) and sand (70%) when the radicles reached approximately 1 cm in length. Finally, they were grown in a temperature-controlled glass greenhouse (28°C, 8 h light/16 h dark). Six-leaf-stage seedlings provided root, stem cortex, and leaf tissues for expression profiling. Concurrently, seeds collected at 14, 19, 25, 29, 35, 41, and 45 days post-pollination (DAP) enabled temporal tracking of *JcLEA* gene expression during seed maturation. Uniformly growing physic nut seedlings (6-leaf stage) were selected for drought and salt stress experiments. Physic nut seedlings were subjected to drought by withholding water. Leaf samples were harvested at 2, 4, and 7 days post-stress, following rapid immersion in liquid nitrogen, tissues were transferred to a -80 °C for archival storage. Salt treatment involved bi-daily irrigation (8:00 AM) with 1 L of Hoagland solution (pH 6.0) supplemented with 100 mM NaCl. Leaf tissues were collected at 2 hours (h), 2 days (d), and 4 days post-treatment, leaf tissues underwent cryofixation via liquid nitrogen immersion (10 sec) prior to storage in a -80 °C cryogenic repository. Raw sequence data under drought (with accession number PRJNA257901) and salt stress (with accession number PRJNA244896) conditions were generated per standardized workflows and subsequently deposited into the NCBI Sequence Read Archive (SRA).

### Gene cloning and transgenic plant construction

Primer 5.0 software engineered the specific primers for the *JcLEA1* gene. Standardized PCR protocols were performed with a cDNA template derived from physic nut roots. The amplified product was subsequently purified and cloned into the pMD18-T vector via TA-cloning. Following transformation, positive clones were identified and verified by sequencing. The confirmed *JcLEA1* sequence was then ligated into the recognition sites of *Kpn* I and *Xba* I endonucleases located downstream of the 35S promoter in the pCAMBIA1301 expression vector using T4 DNA ligase, resulting in the plant expression construct. *Agrobacterium tumefaciens* GV3101 mediated the delivery of recombinant plasmids into Arabidopsis through floral dip transformation (Zhang et al., 2006). Candidate *JcLEA1* transgenic lines were first selected through hygromycin resistance screening, and transformation success was additionally confirmed by transcript-level expression analysis.

### Intracellular distribution of *JcLEA1*

The *JcLEA1* coding region (stop codon excluded) was directionally cloned into the pBWA(V)HS-GlosGFP vector to generate the fusion vector pBWA(V)HS-JcLEA1-GlosGFP. Arabidopsis protoplasts were co-transfected with 35S::JcLEA1-GFP and 35S::GFP constructs via polyethylene glycol transfection (Tang et al., 2019). Localization patterns were captured with a TCS SP8 confocal microscope, following established protoplast isolation protocols (Tang et al., 2019).

### Drought stress treatment

Following surface sterilization, seeds from wild-type and *JcLEA1* transgenic plants were stratified at 4 °C for 2 days to break dormancy. Following surface sterilization, seeds underwent aseptic transfer to 1/2 MS agar plates for vertical cultivation over a 4-day period. Rooted seedlings were then relocated to growth containers filled with a 1:3 (v/v) blend of vermiculite and nutrient-rich substrate. After 15 days of growth under standard chamber conditions (22 ± 1 °C; a 6-hour photoperiod (light) and 8-hour scotophase (dark)), plants were allocated into two distinct groups: with one designated as the control group receiving regular nutrient solution irrigation, while the experimental group underwent drought stress by water withholding. Both groups received identical nutrient supply to ensure differences were solely due to water deficit. The TDR-350 soil moisture probe (Spectrum Technologies) provided daily volumetric water content measurements throughout the experimental period, with drought conditions maintained at 20–25% field capacity until sampling. Survival rate was assessed after drought stress treatment followed by 4 days of rehydration.

### Physiological parameter assessment

Rosette leaves of 15-day drought-exposed and well-watered Arabidopsis plants were harvested and processed for biochemical assays (three independent biological replicates). Relative electrolyte leakage (REL) assay. Freshly excised leaf segments (0.5 cm × 0.5 cm, midribs excluded) were triple-rinsed with deionized water and blotted to remove surface moisture. The discs were then submerged in 10 mL of deionized water at 25°C for 2 h with periodic shaking to enhance electrolyte release. A conductivity meter (METTLER TOLEDO SevenExcellence™ S470) recorded baseline electrolyte levels (C1). Tubes containing leaf discs were then subjected to 15-min boiling, equilibrated to 25°C, and post-treatment conductivity (C2) measured. REL was derived from: REL (%) = (C1/C2)×100. Proline extraction protocol. Leaf tissues (0.1 g FW) were snap-frozen in liquid nitrogen, pulverized to homogeneity, and heated to boiling point (100°C) in a 3% weight/volume sulfosalicylic acid solution for 10 minutes to achieve with continuous agitation. Centrifugal clarification (10,000 ×g, 10 min) yielded supernatant for subsequent processing. A 1 mL aliquot underwent mixing with 2 mL glacial acetic acid and 2 mL acidic ninhydrin reagent (prepared by dissolving 1.25 g ninhydrin in 30 mL acetic acid and 20 mL 6 M phosphoric acid), followed by heating at 100°C for 30 minutes. Following thermal equilibration to ambient conditions, 4 mL toluene was vortex-mixed to extract chromophores. Absorbance readings (520 nm) of the toluene phase were acquired, with proline concentration (μg/g FW) calculated via standard curve interpolation. MDA content and SOD activity were measured as described (Tang et al., 2025). The betaine content was quantified using an ELISA assay, with specific experimental procedures conducted followed the manufacturer-provided guideline.

### RNA isolation and qRT-PCR

Total RNA from physic nut and Arabidopsis tissues was isolated using the Magen Plant RNA Kit (http://magentec.com.cn/) under manufacturer-specified conditions. RNA integrity assessment via 1% agarose gel electrophoresis preceded cDNA synthesis (PrimeScript^TM^ RT Master Mix, TAKARA) for downstream PCR. qRT-PCR was performed with SYBR^®^ Premix Ex Taq^TM^ (TaKaRa, Japan) and the LightCycler® 480 real-time PCR system (Roche; for more details, refer to http://www.roche.com/). The reaction conditions were set as follows: initial denaturation at 95°C for 30 s, then 40 cycles consisting of denaturation at 95°C for 5 s, annealing at 60°C for 20 s, and extension at 72°C for 20 s. Triplicate biological experiments under identical conditions were processed per sample, normalized to the endogenous control *JcActin*. Detailed information regarding primer sequences is presented in **Supplementary Table S1**.

### Statistical analysis

SPSS Statistics version 21.0 facilitated all statistical evaluations, employing ANOVA for group variance assessment, Student's t-tests for pairwise comparisons, and *post hoc* pairwise comparisons were applied using Fisher’s LSD test at α=0.05. An adjusted significance threshold (p-value < 0.01) was applied to account for multiple comparisons.

## Results

### Identification of LEA gene family members in physic nut

To identify LEA family members in physic nut (*Jatropha curcas*), we performed a comprehensive genome-wide search using BLASTP with 51 characterized Arabidopsis and 34 rice LEA proteins as queries. This was complemented by HMMER 3.1 profiling to ensure comprehensive identification. This dual approach identified 24 LEA proteins in physic nut. These proteins were assigned sequential identifiers JcLEA1 to JcLEA24 based on their physical order along chromosomes within linkage groups 1-10, numbered from proximal to distal ends. Key physicochemical parameters of the deduced JcLEA proteins were computed using ExPASy (detailed in **Supplementary Table S2**). Coding sequence lengths varied from 241 bp (JcLEA11) to 1254 bp (JcLEA18), encoding polypeptides of 80–417 residues. Corresponding protein molecular weights ranged from 8.74 kDa (JcLEA11) to 44.80 kDa (JcLEA13), with isoelectric points (pI) spanning 4.61 (JcLEA10) to 10.12 (JcLEA20). Grand average hydropathicity (GRAVY) values were uniformly negative (-1.573 [JcLEA12] to -0.102 [JcLEA19]), confirming high hydrophilicity. Instability indices ranged from 18.34 (JcLEA22) to 76.30 (JcLEA11), with 66.7% (16/24) exhibiting values < 40, indicating most JcLEA proteins are unstable.

### Phylogenetic analysis of LEA proteins

**Figure 1:**
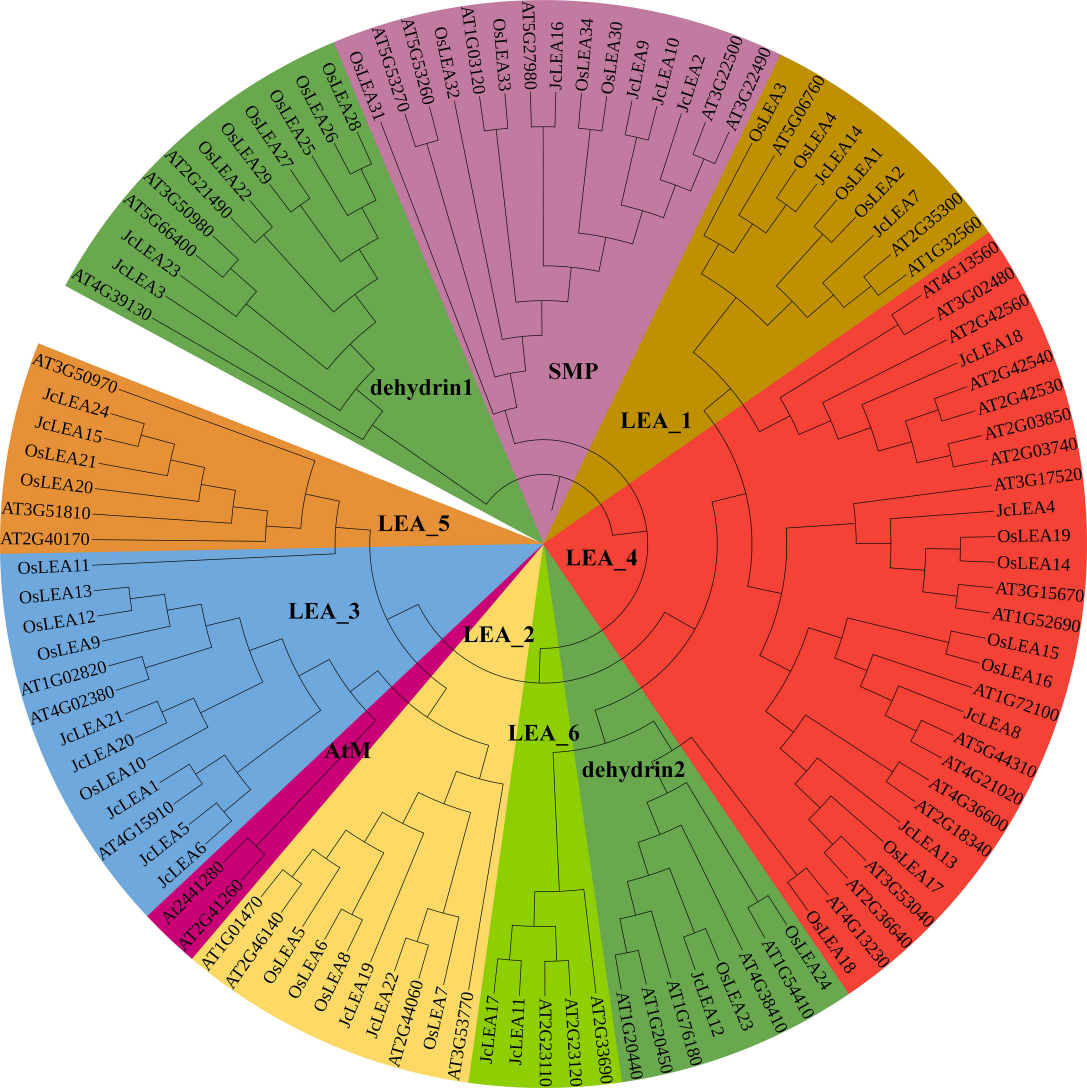
Phylogenetic analysis of 109 LEA proteins (24 from physic nut, 34 from rice, 51 from Arabidopsis) using the Neighbor-Joining method in MEGA 10.0 with 1000 bootstrap replicates. Most branches exhibit bootstrap values ≥70%, confirming the robustness of the clustering.

A phylogenetic tree was constructed using 109 LEA protein sequences (24 physic nut, 34 rice, 51 Arabidopsis) to elucidate evolutionary relationships (**Figure 1**). Consistent with canonical classification in Arabidopsis and rice (Hundertmark et al., 2008; Wang et al., 2007), these proteins divided into nine conserved groups: LEA_1, LEA_2, LEA_3, LEA_4, LEA_5, LEA_6, dehydrin (DHN), seed maturation protein (SMP), and AtM. Among the physic nut proteins: JcLEA7 and JcLEA14 clustered in LEA_1; JcLEA19 and JcLEA22 in LEA_2; JcLEA1, JcLEA5, JcLEA6, JcLEA20, and JcLEA21 in LEA_3; JcLEA4, JcLEA8, JcLEA13, and JcLEA18 in LEA_4; JcLEA15 and JcLEA24 in LEA_5; JcLEA11 and JcLEA17 in LEA_6; JcLEA3, JcLEA12, and JcLEA23 in Dehydrin; JcLEA2, JcLEA9, JcLEA10, and JcLEA16 in SMP. A phylogeny based solely on the 24 JcLEA sequences confirmed these groupings, dividing them into eight subgroups (**Supplementary Figure S1**), supporting the reliability of the cross-species analysis.

### Gene structure analysis of *JcLEA* genes

**Figure 2:**
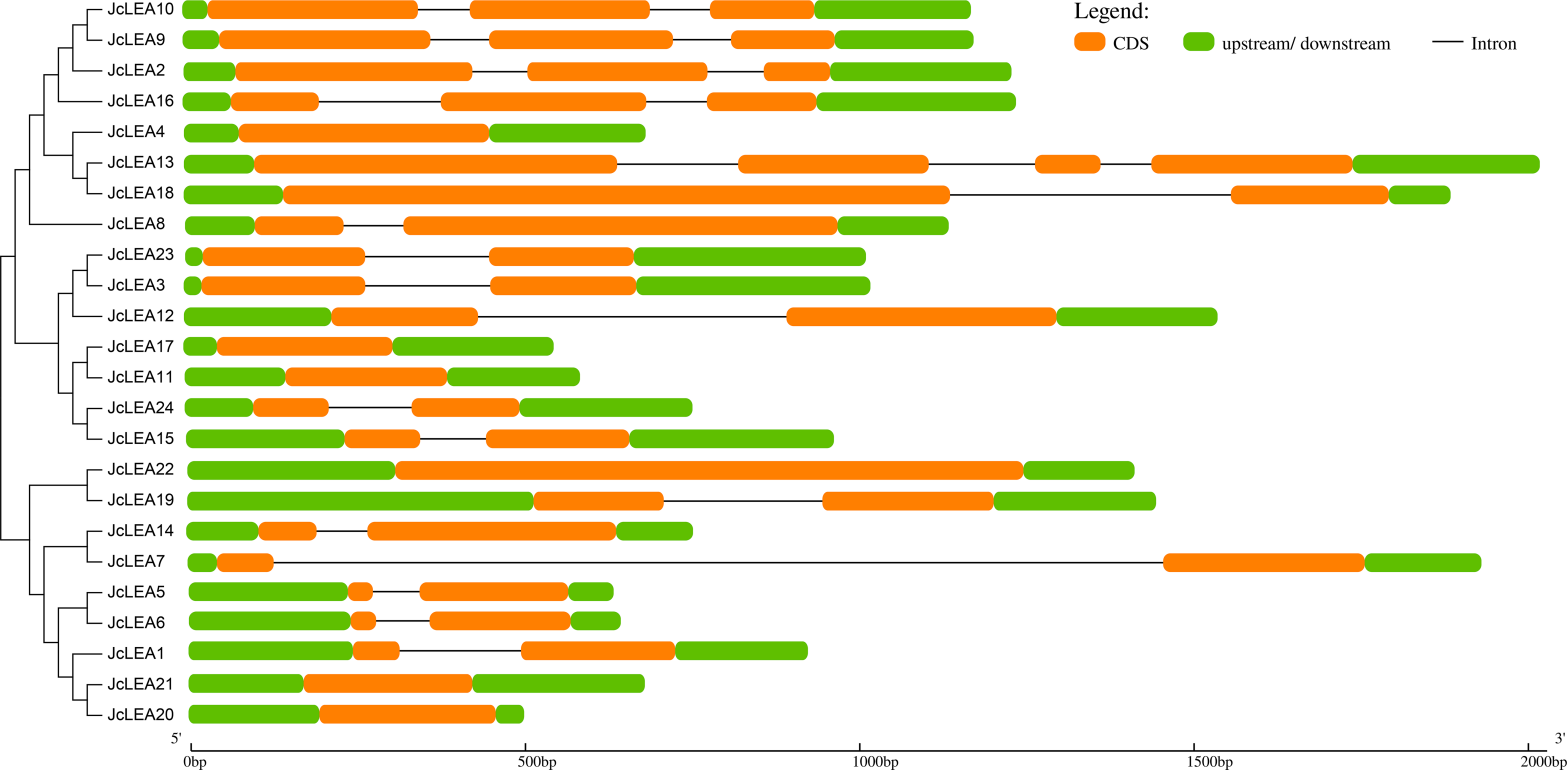
Exon-intron structures of *JcLEA* genes. Exons are depicted as orange rectangular, while introns are indicated by narrow connecting lines, green boxes highlight untranslated regions at both upstream and downstream positions.

Intron/exon structural diversity often drives gene family evolution and provides evidence supporting phylogenetic relationships (Sánchez et al., 2003). Analysis using GSDS revealed relatively simple structures among the 24 *JcLEA* genes, containing 1–4 exons and 0–3 introns (**Figure 2**). Five genes (*JcLEA4*, *JcLEA11*, *JcLEA17*, *JcLEA20*, *JcLEA21*) were intronless, while *JcLEA13* possessed the most introns. Consistent with reports in Arabidopsis and rice (Hundertmark and Hincha, 2008; Wang et al., 2007), genes within the same phylogenetic group generally shared similar structures. For example, SMP group genes (*JcLEA2*, *JcLEA9*, *JcLEA10*, *JcLEA16*) uniformly contained three exons and two introns, while Dehydrin group genes (*JcLEA3*, *JcLEA12*, *JcLEA23*) contained two exons and one intron. This structural conservation within groups further validates the phylogenetic classification.

### Analysis of JcLEA protein conserved motifs

**Figure 3:**
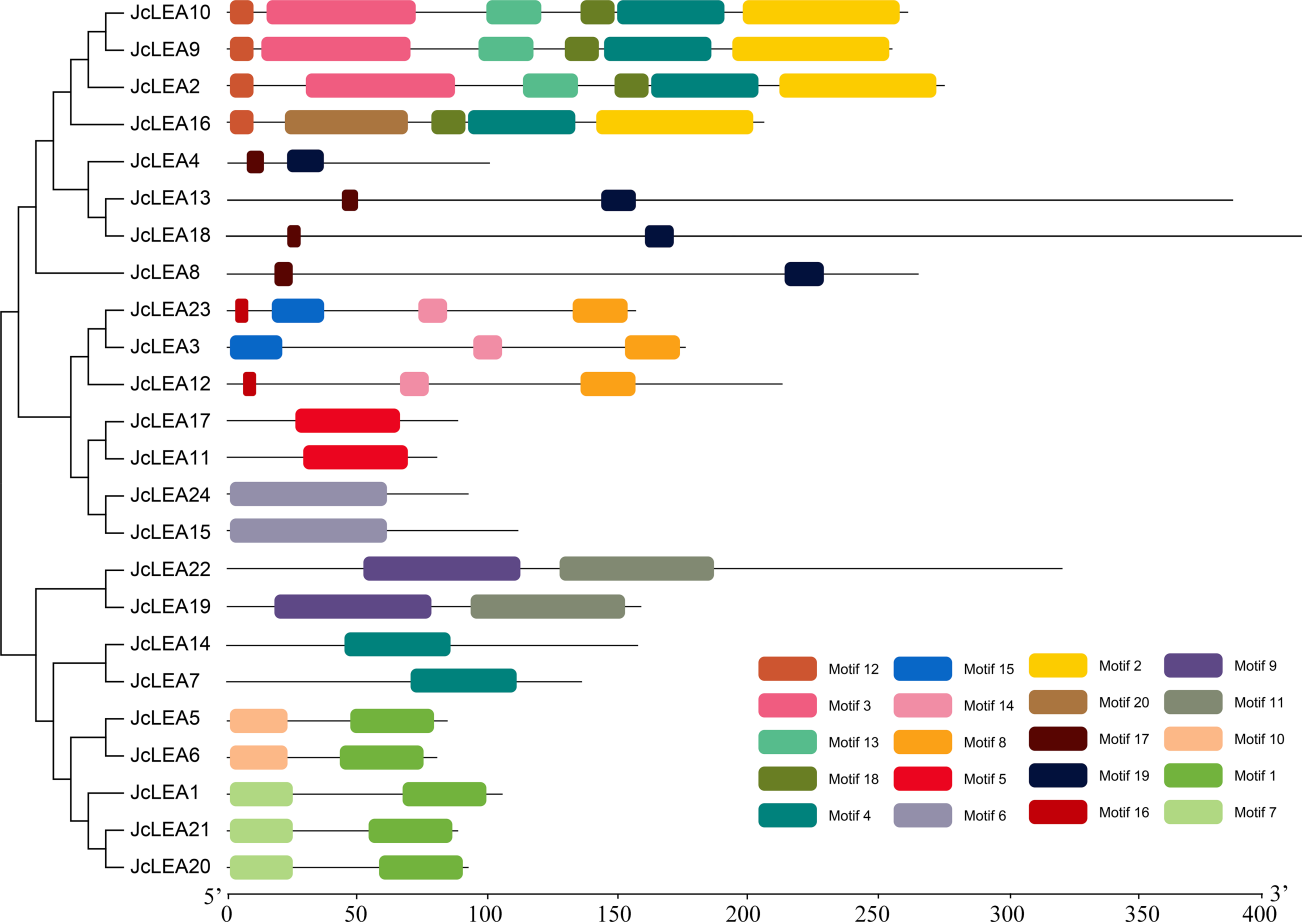
Conserved motifs are distributed in JcLEA proteins. Non-conserved sequences are represented by gray lines, with motif positions indicated by colored boxes.

Conserved motifs provide critical support for phylogenetic grouping. MEME analysis identified 20 conserved motifs within the full-length JcLEA protein sequences (**Figure 3**). Distinct motif compositions characterized each group. For instance, SMP group proteins (JcLEA2, JcLEA9, JcLEA10, JcLEA16) uniquely contained motifs 2, 3, 4, 12, 13, 18, and 20. Motif 5 was exclusive to the LEA_6 group (JcLEA11, JcLEA17). Dehydrin group proteins (JcLEA3, JcLEA12, JcLEA23) uniquely possessed motifs 8, 14, 15, and 16, while motifs 9 and 11 were specific to the LEA_2 group (JcLEA19, JcLEA22). This group-specific motif distribution strongly supports the reliability of the JcLEA protein phylogenetic classification.

### Chromosome localization of JcLEA proteins

**Figure 4:**
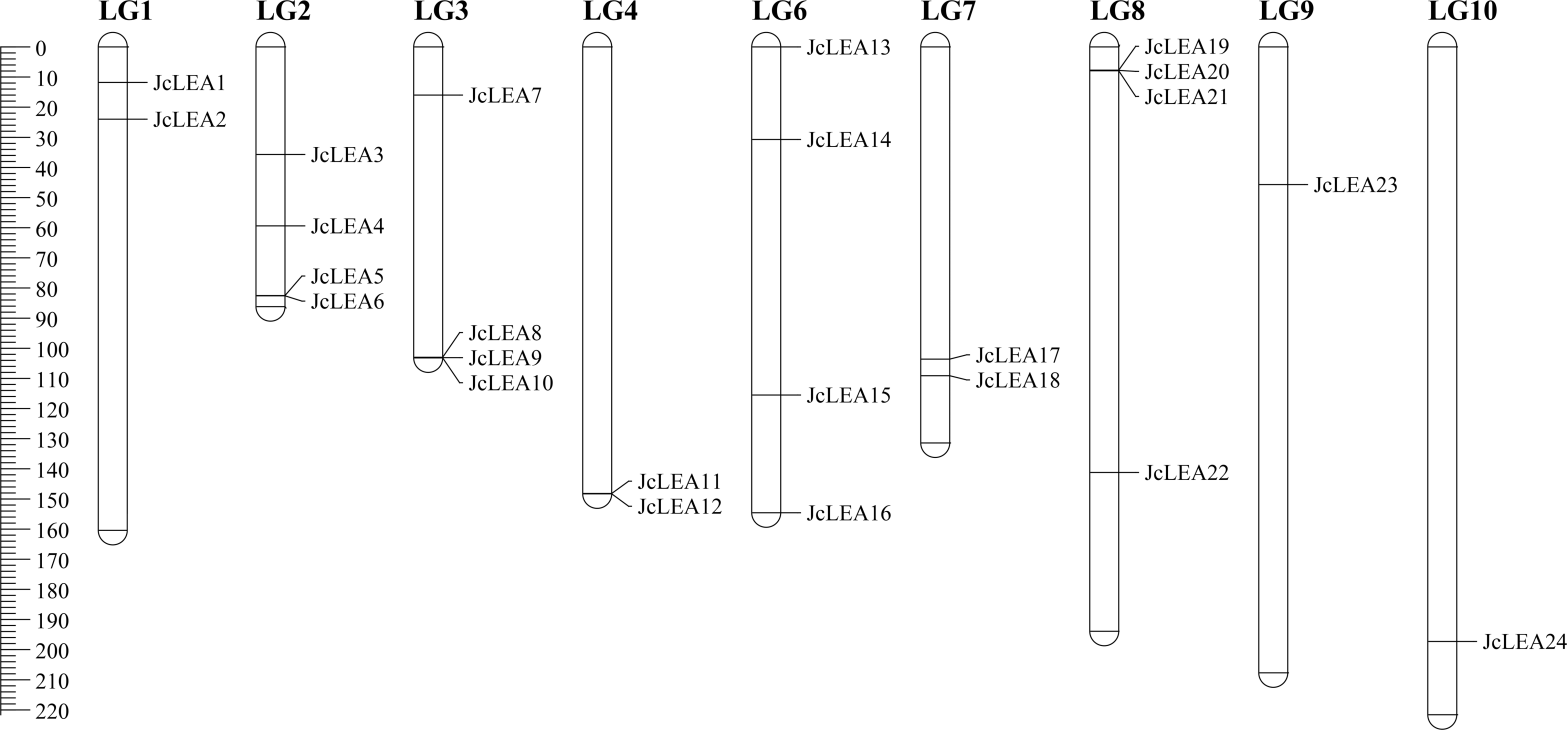
Chromosomal locations of *JcLEA* genes based on the established linkage map. The 24 identified *JcLEA* genes were distributed across nine distinct linkage groups (LGs). Genetic distances are indicated in centiMorgans (cM).

Genomic annotation revealed an uneven distribution of all 24 JcLEA proteins across nine linkage groups (LGs) (**Figure 4**). LG2, LG3, LG6, and LG8 each contained four JcLEA proteins. LG9 and LG10 each contained one JcLEA protein, while LG1, LG4, and LG7 each contained two. No JcLEA proteins were localized to LG5 or LG11. Tandem duplication events, defined as paralogs within 50 kb genomic proximity or separated by ≤3 nonhomologous intervening genes (Cannon et al., 2004), were identified as potential drivers of functional diversification. Three tandem duplication clusters were detected: JcLEA8/JcLEA9/JcLEA10; JcLEA11/JcLEA12; and JcLEA19/JcLEA20/JcLEA21.

### Analysis of JcLEA protein interaction networks

**Figure 5:**
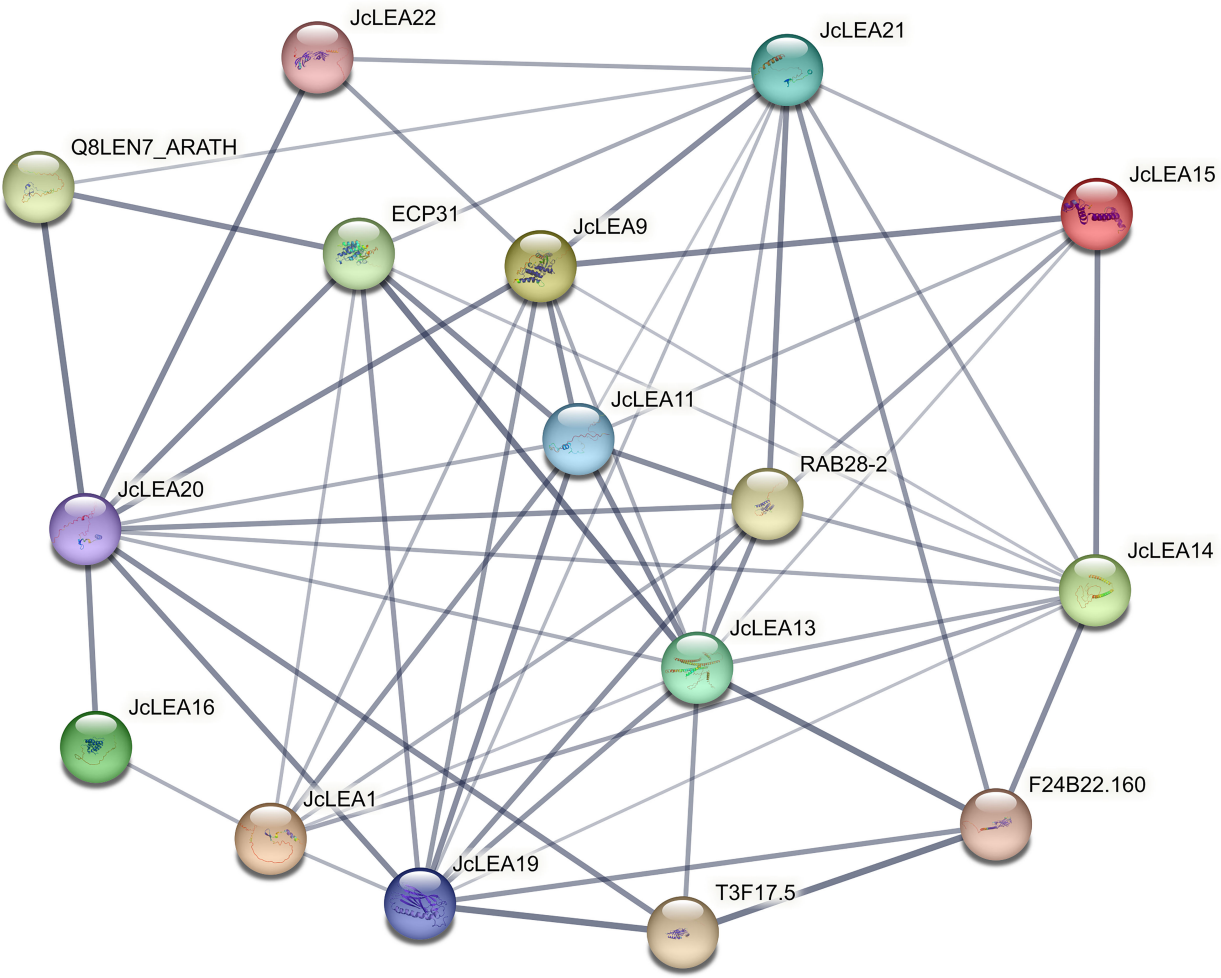
Protein interaction network analysis of JcLEA Proteins. Line thickness indicates the predicted interaction probability between proteins, with thicker lines representing higher confidence in functional association (ranging from low to high based on STRING’s scoring system).

Using Arabidopsis as a reference model, we predicted protein-protein interactions (PPIs) among JcLEA proteins using the STRING database. Eleven JcLEA proteins formed a putative interaction network (**Figure 5**). JcLEA22 was predicted to interact with JcLEA9, JcLEA20, and JcLEA21. JcLEA9 exhibited potential interactions with JcLEA1, JcLEA11, JcLEA13, JcLEA14, JcLEA15, JcLEA19, JcLEA20, and JcLEA21, though interactions with JcLEA1, JcLEA13, and JcLEA14 had lower confidence scores. Homology-based projections suggested JcLEA13 may interact with Arabidopsis proteins EPC31 (high confidence), F24B22.160 (high confidence), RAB28-2, and T3F17.5; JcLEA11 with EPC31 and RAB28-2; and JcLEA20 with LEN7.

### Identification of *cis*-acting elements in *JcLEA* promoters

**Figure 6:**
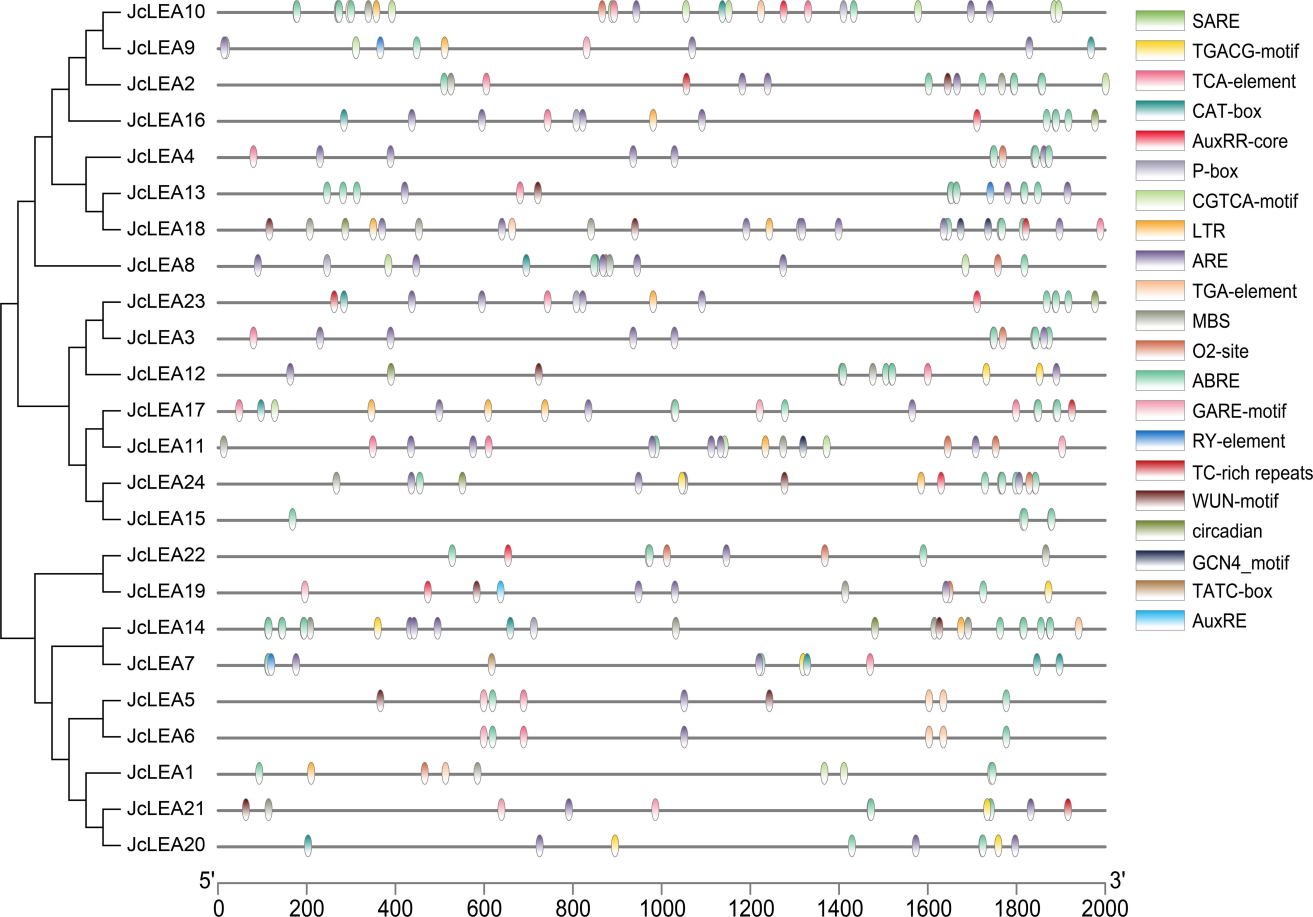
Analysis of cis-regulatory elements within JcLEA promoters in physic nut. Computational prediction was conducted using PlantCARE, with visual annotation implemented via TBtools.

We systematically characterized cis-regulatory elements within 2-kb promoter regions upstream of *JcLEA* genes, revealing core elements (TATA-box, CAAT-box) alongside numerous motifs associated with development, stress adaptation, and hormone responses (**Figure 6**; **Supplementary Table S3**). Developmental elements included the meristem-associated CAT-box (*JcLEA7*, *JcLEA8*, *JcLEA9*, *JcLEA10*, *JcLEA14*, *JcLEA16*, *JcLEA17*, *JcLEA20*, *JcLEA23*), seed metabolism-enriched O2-site (*JcLEA1*, *JcLEA3*, *JcLEA4*, *JcLEA8*, *JcLEA10*, *JcLEA11*, *JcLEA19*, *JcLEA22*, *JcLEA24*), seed-specific RY-element (*JcLEA7*, *JcLEA9*, *JcLEA13*), and widespread circadian rhythm motifs. Hormone-responsive elements featured abundant ABREs (12 copies in *JcLEA14*, 10 in *JcLEA13*), MeJA-responsive motifs (CGTCA/TGACG) in 15 genes, and gibberellin-related elements (GARE-motif/P-box) in 12 genes. Stress-associated elements comprised LTR enrichment (*JcLEA17*: 3 copies; *JcLEA18*: 2 copies), abundant AREs in 22 genes, and MBS sites in 13 genes.

### Expression profiling of *JcLEA* genes in physic nut tissues

**Figure 7:**
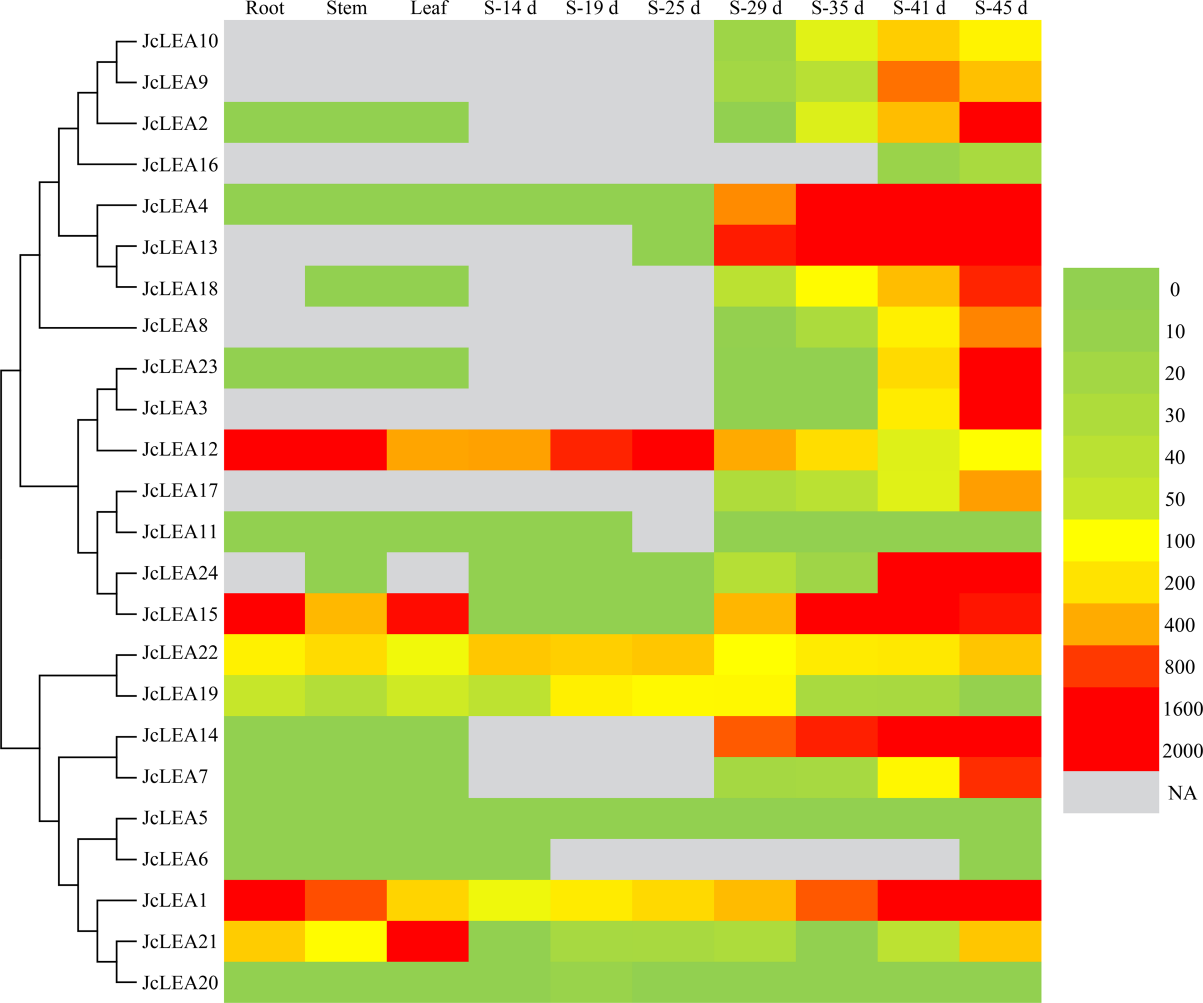
Expression profiles of *JcLEA* genes in tissues (roots, stem cortex, leaves, seeds [S]) of physic nut during distinct developmental phases. The bottom panel displays a color gradient scale correlating with gene expression intensity. NA: not available.

We characterized the spatial expression profiles of *JcLEA* genes across vegetative organs (roots, stem cortex, leaves) and seeds (**Figure 7**; **Supplementary Table S4**). All 24 *JcLEA* genes showed detectable expression in at least one tissue. Most genes exhibited preferential expression in seeds, with pronounced upregulation during late seed maturation stages. Five genes (*JcLEA3*, *JcLEA8*, *JcLEA9*, *JcLEA10*, *JcLEA16*) displayed seed-specific expression, peaking in late maturation. Conversely, five genes (*JcLEA1*, *JcLEA15*, *JcLEA19*, *JcLEA21*, *JcLEA22*) showed consistently high expression in all tissues. Twelve genes (*JcLEA2*, *JcLEA4*, *JcLEA5*, *JcLEA6*, *JcLEA7*, *JcLEA13*, *JcLEA14*, *JcLEA17*, *JcLEA18*, *JcLEA20*, *JcLEA23*, *JcLEA24*) exhibited low expression (<0.5 TPM) in vegetative tissues; however, nine of these (*JcLEA2*, *JcLEA4*, *JcLEA7*, *JcLEA13*, *JcLEA14*, *JcLEA17*, *JcLEA18*, *JcLEA23*, *JcLEA24*) showed high expression (>0.5 TPM) in seeds. During seed development, nine genes (*JcLEA2*, *JcLEA3*, *JcLEA4*, *JcLEA7*, *JcLEA8*, *JcLEA14*, *JcLEA18*, *JcLEA23*, *JcLEA24*) peaked at 45 days after pollination (DAP), while *JcLEA12* peaked at 25 DAP.

### Transcriptional response of *JcLEA* genes to abiotic stress

**Figure 8:**
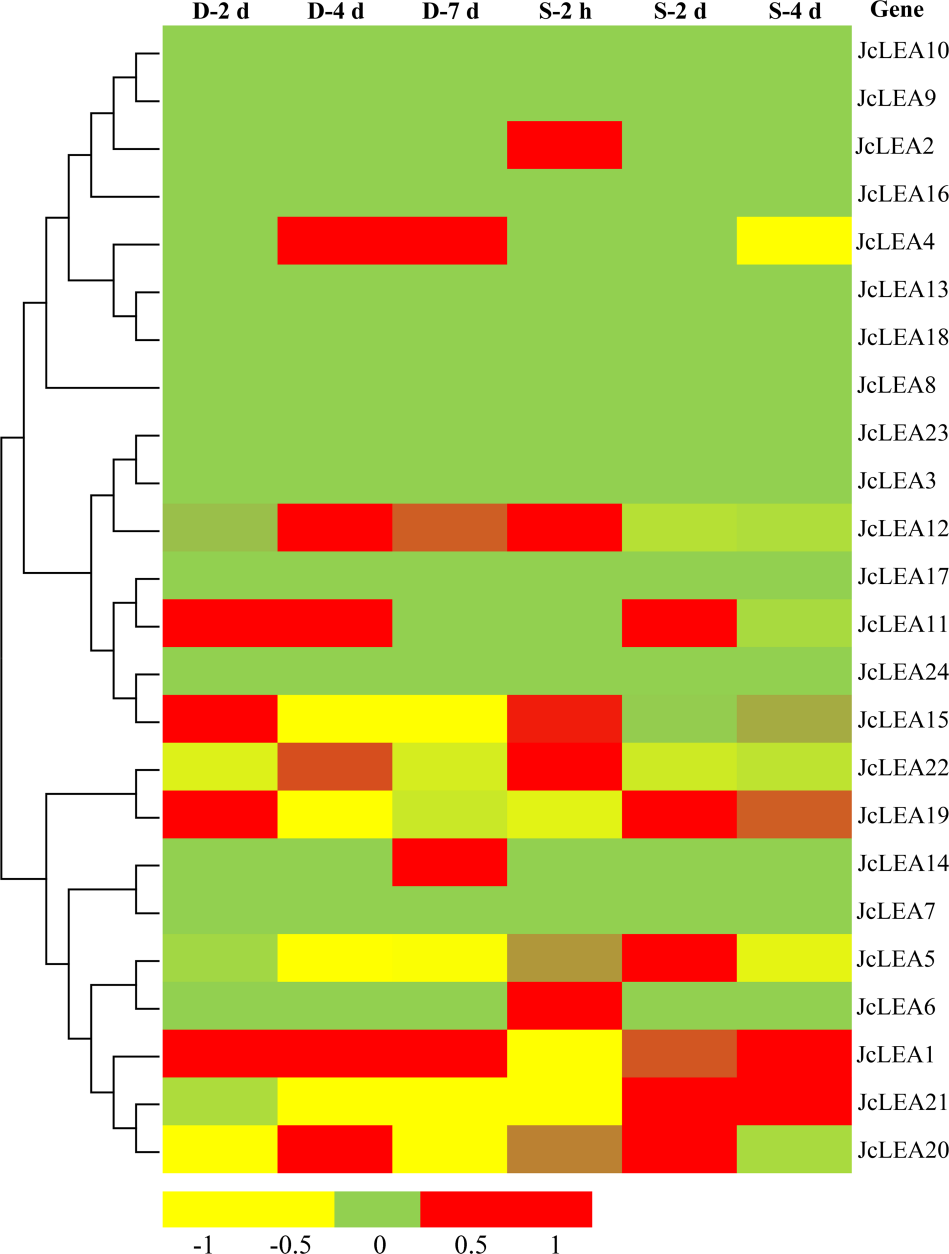
Transcriptional analysis of 24 *JcLEA* genes in root tissues of physic nut subjected to drought and salinity stress. Heatmap displays log_2_-transformed ratios of treated vs. control samples (RNA-seq data), with expression level color scale at the bottom.

RNA-seq analysis of physic nut roots under drought and salinity stress revealed that 13 *JcLEA* genes responded significantly (adjusted p-value < 0.05) to at least one stress treatment at specific time points, while 11 genes showed no significant change (**Figure 8**). Among responsive genes, nine (*JcLEA1*, *JcLEA4*, *JcLEA6*, *JcLEA11*, *JcLEA12*, *JcLEA15*, *JcLEA19*, *JcLEA20*, *JcLEA21*) exhibited fold-changes > |2| (log_2_ scale). *JcLEA1*, *JcLEA4*, and *JcLEA21* showed particularly strong induction under drought. *JcLEA1* displayed sustained upregulation across all drought time points.

### Subcellular localization of the JcLEA1 protein

**Figure 9:**
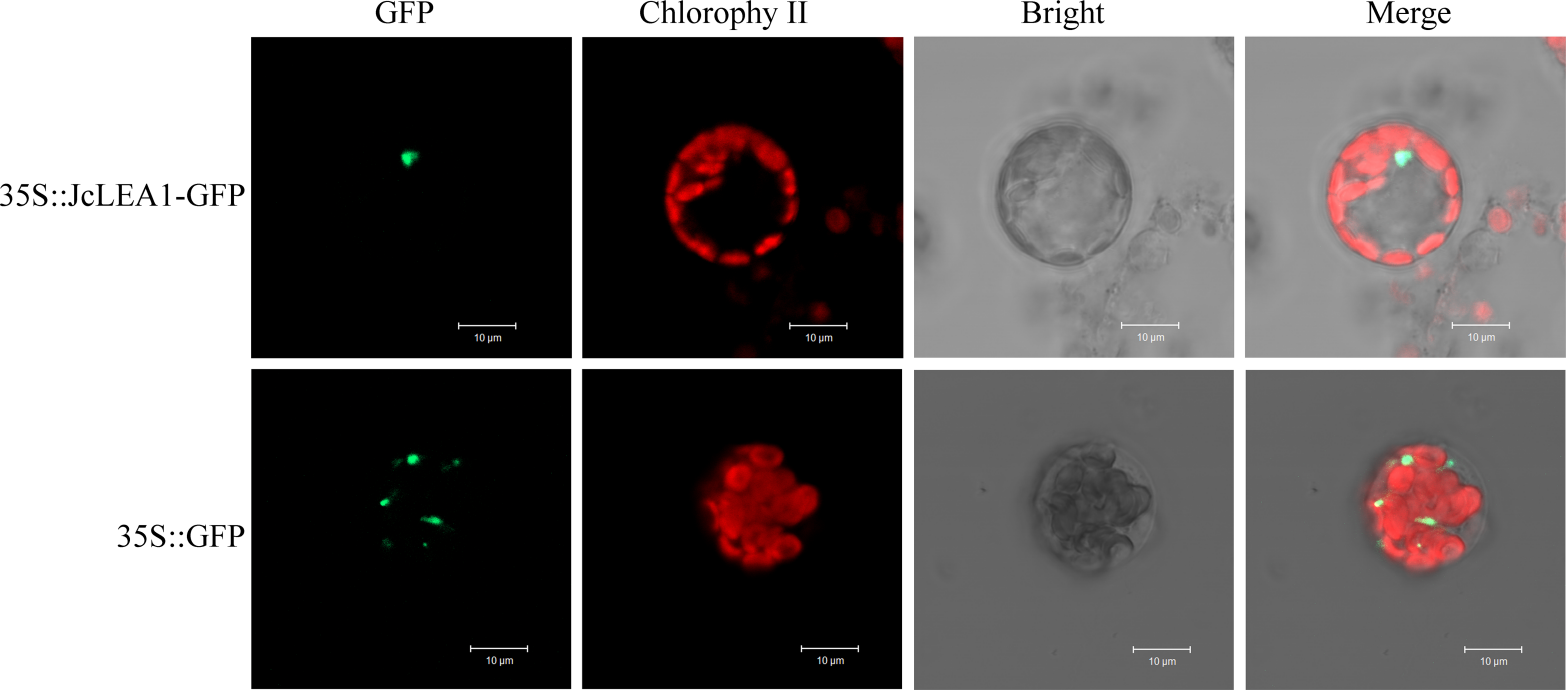
Subcellular localization of JcLEA1 protein.

To investigate JcLEA1 protein localization, we performed transient expression assays in Arabidopsis protoplasts using polyethylene glycol (PEG)-mediated transfection with two constructs: JcLEA1-GFP and empty GFP vector as control. Confocal microscopy analysis revealed distinct distribution patterns between the two groups. Specifically, the JcLEA1-GFP fusion protein exhibited exclusive nuclear accumulation, while GFP fluorescence in control cells displayed diffuse cytoplasmic localization throughout the entire cell (**Figure 9**). This observation confirms that the JcLEA1 protein is nuclear-localized.

### *JcLEA1* enhances drought tolerance in Arabidopsis

**Figure 10:**
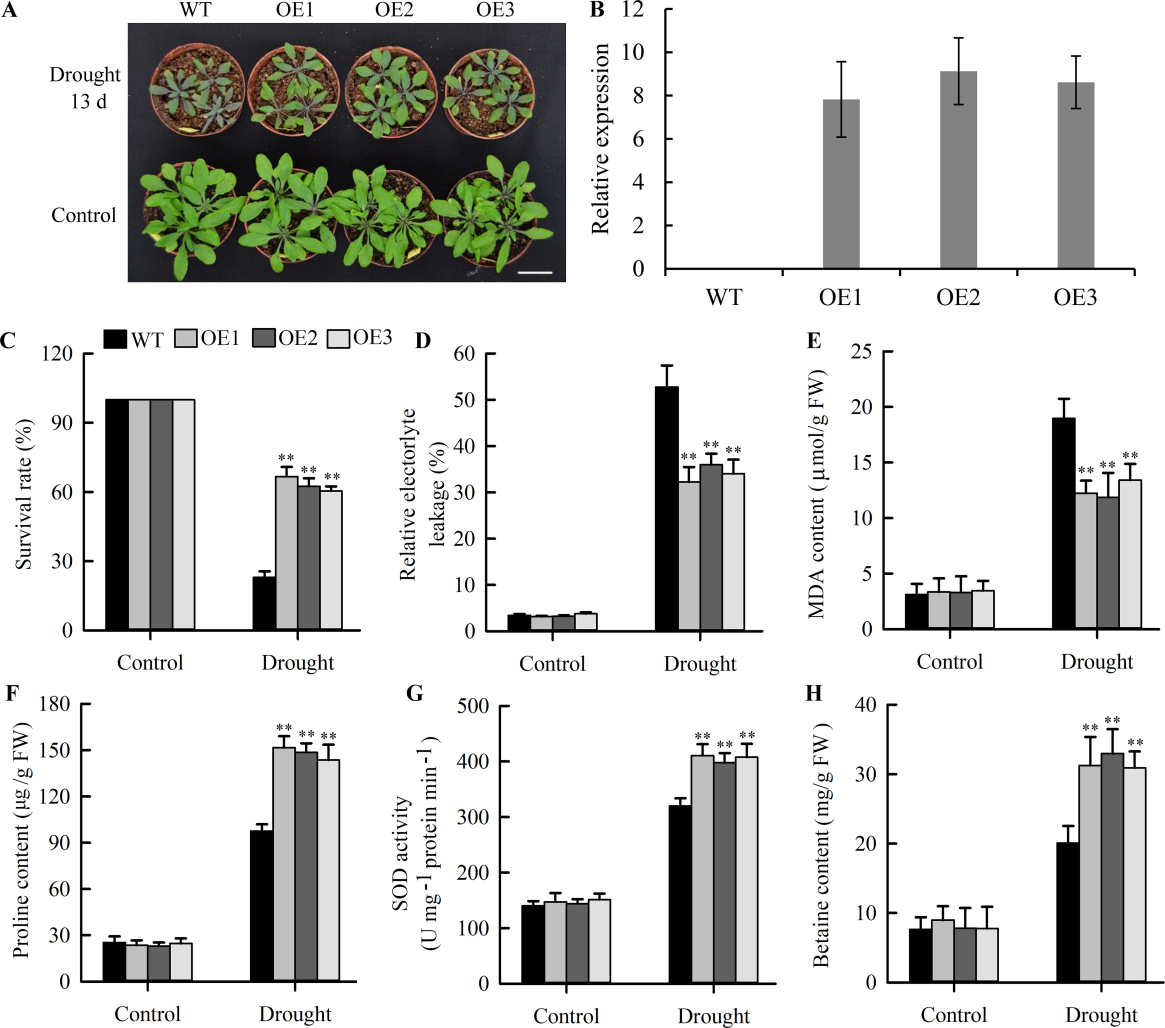
Drought tolerance analysis of wild-type (WT) and *JcLEA1*-overexpressing Arabidopsis. **(A)** Phenotypic responses under drought and control conditions (representative images from triplicate independent biological replicates), scale bars:1 cm; **(B)** Transcript abundance of *JcLEA1* in WT and transgenic lines; **(C)** Survival rates of WT and *JcLEA1*-overexpressing Arabidopsis. **(D–H)** Physiological stress indicators in leaves: **(D)** Relative electrolyte leakage (REL), **(E)** MDA content, **(F)** proline content, **(G)** SOD activity, and **(H)** betaine levels measured before and after stress treatment. Data in C-H: Values represent means ± SD (n=20 plants per genotype) from three independent experiments. Significance thresholds were defined as p < 0.01 (Student’s t-test) relative to WT controls, with double asterisks highlighting these differences.

To validate the drought responsiveness of *JcLEA* genes identified by transcriptomics, we generated *JcLEA1*-overexpressing transgenic Arabidopsis lines via *Agrobacterium*-mediated transformation. Quantitative RT-PCR confirmed significantly higher *JcLEA1* transcript levels in transgenic lines compared to wild-type (WT) controls, where no expression was detected in WT plants (**Figure 10B**).

Under well-watered conditions, transgenic and WT plants showed indistinguishable phenotypes. Following water deficit stress, *JcLEA1*-overexpressing plants exhibited larger rosettes, reduced chlorophyll loss, and less severe growth inhibition than WT (**Figure 10A**). Furthermore, transgenic plants displayed significantly reduced electrolyte leakage (**Figure 10C**) and higher survival rates (**Figure 10D**) under drought stress. Physiological assays revealed no significant differences in malondialdehyde (MDA) content, proline accumulation, superoxide dismutase (SOD) activity, or glycine betaine content between unstressed transgenic and WT plants. Critically, however, under drought stress, *JcLEA1*-overexpressing plants showed significantly lower MDA (**Figure 10E**), higher proline (**Figure 10F**), elevated SOD activity (**Figure 10G**), and increased glycine betaine (**Figure 10H**) compared to stressed WT controls.

These observed drought-resistant phenotypes and coordinated physiological improvements directly correlated with the high induction of *JcLEA1* expression under dehydration in the original RNA-seq data. Thus, this functional validation not only confirms the accuracy of the transcriptomic analysis for LEA family genes but also establishes *JcLEA1* as a key regulator of plant drought adaptation.

## Discussion

The LEA proteins, which function as important regulators in plant growth, development, and stress responses, remain poorly characterized in non-model species such as the drought-tolerant bioenergy crop physic nut. This shrub’s extensive root system, water-use efficiency, and drought resilience make it an ideal model for studying plant adaptation to water deficit (Openshaw, 2000). In this study, we performed phylogenetic analyses, conserved motif identification, expression profiling and functional analysis of *JcLEA* genes. Heterologous expression of *JcLEA1* enhanced drought tolerance in Arabidopsis, supporting the functional involvement of *JcLEA* genes in drought stress responses. Our findings provide insights into LEA functions in developmental processes and stress adaptation of physic nut and are consistent with the conserved cytoprotective role of *LEA* genes in plant abiotic stress adaptation.

We identified twenty-four *JcLEA* genes in physic nut (320 Mb genome), fewer than the 34 *OsLEA* genes in rice (466 Mb genome) and 51 *AtLEA* genes in Arabidopsis (125 Mb genome) (Hundertmark and Hincha, 2008; Wang et al., 2007; Wu et al., 2015; Yu et al., 2002). This discrepancy may be attributable to lineage-specific gene losses during evolution. As a stress-tolerant species, physic nut may exhibit selective retention of functionally critical LEA subtypes with potential elimination of redundant paralogs (Wu et al., 2015). Notably, this streamlined gene family architecture persists despite tandem duplication events, contrasting with the extensive LEA family expansions in rice and Arabidopsis (Hundertmark and Hincha, 2008; Wang et al., 2007). This evolutionary dynamic is consistent with previous findings suggesting that stress-adapted species often maintain compact stress-response gene families through paralog pruning (Ashraf and Foolad, 2007).

Our phylogenetic analysis further revealed that the 24 JcLEA proteins were distributed across eight of the nine canonical LEA subgroups (LEA_1 to LEA_6, DHN, SMP), with no members clustering in the AtM subgroup (**Figure 1**). This observation aligns with the unique evolutionary origin of the AtM subgroup, which are initially identified as an Arabidopsis-specific clade with distinct sequence characteristics not universally conserved across plant lineages (Hundertmark and Hincha, 2008). Subsequent studies have confirmed that AtM homologs are absent in several monocot and dicot species, including rice (Wang et al., 2007) and poplar (Cheng et al., 2021), suggesting that AtM may represent a lineage-specific expansion or functional specialization restricted to certain Brassicaceae species. The absence of the AtM subgroup in physic nut may reflect functional redundancy or lineage-specific adaptive evolution. Hundertmark and Hincha (2008) proposed that AtM proteins lack the canonical hydrophilic motifs of other LEA subgroups and may have specialized roles in Arabidopsis-specific stress responses or developmental processes that are non-essential for physic nut’s adaptation to marginal, arid environments.

The subfamily-specific distribution of conserved motifs may reflect functional specialization among physic nut LEA proteins. Unique motifs in SMP (e.g., Motifs 2,3,4) and dehydrin subfamilies (e.g., Motifs 8,14) are positionally consistent with their roles in seed maturation and dehydration tolerance, though further investigation is needed. Similar motif distributions occur in Arabidopsis, rice, and maize (Hundertmark and Hincha, 2008; Wang et al., 2007), providing molecular support for phylogenetic grouping and potentially reflecting divergent evolutionary trajectories driven by functional specialization.

Phylogenetic analysis grouped JcLEA proteins into eight conserved subfamilies (**Figure 1**), demonstrating evolutionary conservation across plant species. Expression profiling revealed that most *JcLEA* genes, particularly SMP members, showed peak expression during late seed maturation (**Figure 7**), consistent with their canonical roles in protecting cellular structures during desiccation in cotton and Arabidopsis (Hundertmark and Hincha, 2008; Tain et al., 2025). The seed-specific expression of *JcLEA3*, *JcLEA8*, *JcLEA9*, *JcLEA10*, and *JcLEA16* suggests involvement in maintaining seed viability under dehydration, a trait contributing to physic nut’s arid adaptation.

RNA-seq analyses have revealed that abiotic stress can induce the upregulation or downregulation of specific *LEA* genes in plant species (Xu M and WANG, 2020). Comparative studies have demonstrated that 24 poplar *LEA* genes are differentially expressed under imposed salt and drought conditions (Cheng et al., 2021), whereas tomato plants show regulation of 10 *LEA* genes in response to similar stressors (Jia et al., 2022). Notably, rapeseed exhibits drought/salinity-mediated modulation of most LEA family members (Wang et al., 2024b), highlighting conserved stress-responsive patterns across divergent taxa. In addition, overexpression or knockout of some *LEA* genes confers enhanced tolerance to environmental adversities such as water deficit and ionic stress, in plants (Wang et al., 2014). For example, heterologous expression of *SiLEA14* confers Arabidopsis higher drought resilience and salt stress adaptation (Wang et al., 2014), and cotton plants genetically modified with *GhLEA3* expression demonstrate improved water deficit and high salinity resistance (Shiraku et al., 2022). While physic nut exhibits documented drought/salinity tolerance (Openshaw, 2000), LEA functions remain poorly characterized. Our RNA-seq data revealed dynamic expression of *JcLEA* genes during drought and/or salinity (**Figure 8**), suggesting their potential function in osmotic stress adaptation. Further transgenic studies are needed to elucidate their biological roles in stress tolerance.

Transgenic plants overexpressing *JcLEA1* exhibited characteristic stress tolerance phenotypes including larger leaves and reduced pigment accumulation (**Figure 10A**), demonstrating that *JcLEA1* overexpression confers protection against cellular dehydration and oxidative stress. As established mechanisms, LEA proteins exert their protective effects by acting as molecular chaperones that stabilize membrane architecture and protein structures during desiccation (Mohanty and Hembram, 2025). MDA is an end-product of polyunsaturated fatty acid peroxidation, and its elevated content is generally regarded as an indicator of oxidative damage to cell membranes (Morales and Munné-Bosch, 2019). Notably, under drought stress, the significantly lower levels of relative electrolyte leakage and MDA content in *JcLEA1* transgenic lines (**Figures 10D** and E), directly supporting *JcLEA1*’s role in protecting membrane integrity—a conclusion reinforced by the established reliability of these parameters as stress-response biomarkers. These findings align with prior studies showing that LEA proteins mitigate lipid peroxidation and stabilize membrane fluidity during water scarcity (Mohanty and Hembram, 2025; Zhou et al., 2023). Together, these results underscore the functional conservation of LEA proteins in maintaining cellular homeostasis across phylogenetically divergent species.

Proline, a key osmoregulatory substance, protects plants from abiotic stresses by maintaining cellular turgor pressure while preserving protein integrity and membrane stability (Szabados and Savouré, 2010). Meanwhile, SOD, a critical antioxidant enzyme, actively scavenges harmful free radicals during oxidative stress (Wang et al., 2005). Under drought stress, *JcLEA1*-transgenic plants exhibited a marked increase in proline accumulation and SOD activity (**Figures 10F** and G), suggesting enhanced capacity to mitigate ROS damage. A similar phenomenon has been observed in *OsLEA4*-overexpressing rice, *GiLEA5-2.1*-expressing tobacco and *GhLEA3*-expressing cotton (Hu et al., 2016; Shiraku et al., 2022; Zhang et al., 2024). The enhanced accumulation of betaine in *JcLEA1*-transgenic plants highlights betaine’s critical role in conferring drought tolerance. As a compatible solute, betaine stabilizes cellular osmotic equilibrium, preserves membrane integrity, and scavenges reactive oxygen species, consistent with its well-characterized drought-resistance mechanisms (Ashraf and Foolad, 2007). The association between betaine accumulation and improved drought resilience suggests its contribution to the transgenic phenotype.

Collectively, this work provides foundational insights into LEA protein phylogeny, structural diversity, and functions in physic nut. While *JcLEA1* validation confirms drought tolerance involvement, these results highlight the need for comprehensive functional characterization of other *JcLEA* candidates through transgenic approaches and stress physiology analyses.

## Conclusions

We have identified 24 full-length *JcLEA* genes, which can be robustly assigned to eight phylogenetic groups. Their expression profiles clearly indicate that some *JcLEA* genes are involved in responses to abiotic stresses. Transgenic expression of one of the genes (*JcLEA1*) enhanced the tolerance of Arabidopsis plants to drought stress, corroborating the hypothesis that some of these genes participate in physic nut’s responses to abiotic stresses. In summary, our results identify candidate genes for future functional analysis of *JcLEA* genes involved in drought-related signaling pathways. They also provide indications of the phylogeny, structural features, and functions of *LEA* genes in physic nut, but much further analysis is required.

## Data Availability

The original contributions presented in the study are included in the article/**Supplementary Material**. Further inquiries can be directed to the corresponding author.

## References

[B1] Ashraf M. Foolad M. R. (2007). Roles of glycine betaine and proline in improving plant abiotic stress resistance. Environ. Exp. Bot. 59, 206–216. doi: 10.1016/j.envexpbot.2005.12.006

[B2] Cannon S. B. Mitra A. Baumgarten A. Young N. D. May G. (2004). The roles of segmental and tandem gene duplication in the evolution of large gene families in *Arabidopsis thaliana*. BMC Plant Biol. 4, 10. doi: 10.1186/1471-2229-4-10, PMID: 15171794 PMC446195

[B3] Chen C. Wu Y. Li J. Wang X. Zeng Z. Xu J. . (2023). TBtools-II: A “one for all, all for one” bioinformatics platform for biological big-data mining. Mol. Plant. 16, 1733–1742. doi: 10.1016/j.molp.2023.09.010, PMID: 37740491

[B4] Cheng Z. Zhang X. Yao W. Zhao K. Liu L. Fan G. . (2021). Genome-wide search and structural and functional analyses for late embryogenesis-abundant (LEA) gene family in poplar. BMC Plant Biol. 21, 110. doi: 10.1186/s12870-021-02872-3, PMID: 33627082 PMC7903804

[B5] Dure L. III Greenway S. C. Galau G. A. (1981). Developmental biochemistry of cottonseed embryogenesis and germination: changing messenger ribonucleic acid populations as shown by *in vitro* and *in vivo* protein synthesis. Biochemistry. 20, 4162–4168. doi: 10.1021/bi00517a033, PMID: 7284317

[B6] Gasteiger E. Gattiker A. Hoogland C. Ivanyi I. Appel R. D. Bairoch A. (2003). ExPASy: the proteomics server for in-depth protein knowledge and analysis. Nucleic Acids Res. 31, 3784–3788. doi: 10.1093/nar/gkg563, PMID: 12824418 PMC168970

[B7] Hu T. Zhu S. Tan L. Qi W. He S. Wang G. (2016). Overexpression of *OsLEA4* enhances drought, high salt and heavy metal stress tolerance in transgenic rice (*Oryza sativa* L.). Environ. Exp. Bot. 123, 68–77. doi: 10.1016/j.envexpbot.2015.10.002

[B8] Hundertmark M. Hincha D. K. (2008). LEA (late embryogenesis abundant) proteins and their encoding genes in *Arabidopsis thaliana*. BMC Genomics. 9, 118. doi: 10.1186/1471-2164-9-118, PMID: 18318901 PMC2292704

[B9] Jia C. Guo B. Wang B. Li X. Yang T. Li N. . (2022). The LEA gene family in tomato and its wild relatives: genome-wide identification, structural characterization, expression profiling, and role of *SlLEA6* in drought stress. BMC Plant Biol. 22, 596. doi: 10.1186/s12870-023-04052-x, PMID: 36536303 PMC9762057

[B10] Jia H. Wang X. Shi Y. Wu X. Wang Y. Liu J. . (2020). Overexpression of *Medicago sativa* LEA 4–4 can improve the salt, drought, and oxidation resistance of transgenic Arabidopsis. PloS One. 15, e0234085. doi: 10.1371/journal.pone.0234085, PMID: 32497140 PMC7272090

[B11] Lei S. Yin J. Li C. Xu Q. Tian B. Cheng X. . (2024). Impacts of natural variations in the *TaLEA-1A* gene on seed dormancy and germination in wheat and transgenic Arabidopsis and rice. Environ. Exp. Bot. 220, 105715. doi: 10.1016/j.envexpbot.2024.105715

[B12] Letunic I. Bork P. (2018). 20 years of the SMART protein domain annotation resource. Nucleic Acids Res. 46, D493–D496. doi: 10.1093/nar/gkx922, PMID: 29040681 PMC5753352

[B13] Ma F. Song S. Li C. Huang D. Wu B. Xing W. . (2024). Passion fruit HD-ZIP genes: characterization, expression variance, and overexpression *PeHB31* enhanced drought tolerance via lignin pathway. Int. J. Biol. Macromol. 276, 133603. doi: 10.1016/j.ijbiomac.2024.133603, PMID: 38969043

[B14] Mohanty S. Hembram P. (2025). An overview of LEA genes and their importance in combating abiotic stress in rice. Plant Mol. Biol. Rep. 43, 337–351. doi: 10.1007/s11105-024-01468-z

[B15] Morales M. Munné-Bosch S. (2019). Malondialdehyde: facts and artifacts. Plant Physiol. 180, 1246–1250. doi: 10.1104/pp.19.00405, PMID: 31253746 PMC6752910

[B16] Openshaw K. (2000). A review of *Jatropha curcas*: an oil plant of unfulfilled promise. Biomass Bioenerg. 19, 1–15. doi: 10.1016/S0961-9534(00)00019-2

[B17] Sánchez D. Ganfornina M. D. Gutiérrez G. Marín A. (2003). Exon-intron structure and evolution of the Lipocalin gene family. Mol. Biol. Evol. 20, 775–783. doi: 10.1093/molbev/msg079, PMID: 12679526

[B18] Shiraku M. L. Magwanga R. O. Zhang Y. Hou Y. Kirungu J. N. Mehari T. G. . (2022). Late embryogenesis abundant gene LEA3 (Gh_A08G0694) enhances drought and salt stress tolerance in cotton. Int. J. Biol. Macromol. 207, 700–714. doi: 10.1016/j.ijbiomac.2022.03.110, PMID: 35341886

[B19] Su M. Huang G. Zhang Q. Wang X. Li C. Tao Y. . (2016). The LEA protein, ABR, is regulated by ABI5 and involved in dark-induced leaf senescence in *Arabidopsis thaliana*. Plant Sci. 247, 93–103. doi: 10.1016/j.plantsci.2016.03.009, PMID: 27095403

[B20] Szabados L. Savouré A. (2010). Proline: a multifunctional amino acid. Trends Plant Sci. 15, 89–97. doi: 10.1016/j.tplants.2009.11.009, PMID: 20036181

[B21] Tang Y. Bao X. Zhi Y. Wu Q. Guo Y. Yin X. . (2019). Overexpression of a MYB family gene, *OsMYB6*, increases drought and salinity stress tolerance in transgenic rice. Front. Plant Sci. 10. doi: 10.3389/fpls.2019.00168, PMID: 30833955 PMC6387972

[B22] Tang Y. Wang X. Wang Y. Xie J. Zhang R. Liu T. . (2025). Heterologous expression of physic nut *JcHDZ25* confers tolerance to drought stress in transgenic rice. BMC Genomics 26, 366. doi: 10.1186/s12864-025-11566-1, PMID: 40217467 PMC11992789

[B23] Tain C. Rehman A. Wang X. Wang Z. Li H. Ma J. . (2025). Late embryogenesis abundant gene *GhLEA-5* of semi-wild cotton positively regulates salinity tolerance in upland cotton. Gene 949, 149372. doi: 10.1016/j.gene.2025.149372, PMID: 40023341

[B24] Wang K. Guo H. Yin Y. (2024a). AP2/ERF transcription factors and their functions in Arabidopsis responses to abiotic stresses. Environ. Exp. Bot. 222, 105763. doi: 10.1016/j.envexpbot.2024.105763

[B25] Wang M. Li P. Li C. Pan Y. Jiang X. Zhu D. . (2014). *SiLEA14*, a novel atypical LEA protein, confers abiotic stress resistance in foxtail millet. BMC Plant Biol. 14, 290. doi: 10.1186/s12870-014-0290-7, PMID: 25404037 PMC4243736

[B26] Wang W. Liu Y. Kang Y. Liu W. Li S. Wang Z. . (2024b). Genome-wide characterization of LEA gene family reveals a positive role of BnaA.LEA6.a in freezing tolerance in rapeseed (*Brassica napus* L.). BMC Plant Biol. 24, 433. doi: 10.1186/s12870-024-05111-7, PMID: 38773359 PMC11106994

[B27] Wang F.-Z. Wang Q.-B. Kwon S.-Y. Kwak S.-S. Su W.-A. (2005). Enhanced drought tolerance of transgenic rice plants expressing a pea manganese superoxide dismutase. J. Plant Physiol. 162, 465–472. doi: 10.1016/j.jplph.2004.09.009, PMID: 15900889

[B28] Wang Z. Zhang Q. Qin J. Xiao G. Zhu S. Hu T. (2021). *OsLEA1a* overexpression enhances tolerance to diverse abiotic stresses by inhibiting cell membrane damage and enhancing ROS scavenging capacity in transgenic rice. Funct. Plant Biol. 48, 860–870. doi: 10.1071/FP20231, PMID: 33820598

[B29] Wang X.-S. Zhu H.-B. Jin G.-L. Liu H.-L. Wu W.-R. Zhu J. (2007). Genome-scale identification and analysis of LEA genes in rice (*Oryza sativa* L.). Plant Sci. 172, 414–420. doi: 10.1016/j.plantsci.2006.10.004

[B30] Wu P. Zhou C. Cheng S. Wu Z. Lu W. Han J. . (2015). Integrated genome sequence and linkage map of physic nut (*Jatropha curcas* L.), a biodiesel plant. Plant J. 81, 810–821. doi: 10.1111/tpj.12761, PMID: 25603894

[B31] Xu M T. Q. WANG Y. (2020). Transcriptomic analysis of the grapevine LEA gene family in response to osmotic and cold stress reveals a key role for VamDHN3. Plant Cell Physiol. 61, 775–786. doi: 10.1093/pcp/pcaa004, PMID: 31967299 PMC10199170

[B32] Yu J. Hu S. Wang J. Wong G. K.-S. Li S. Liu B. . (2002). A draft sequence of the rice genome (Oryza sativa L. ssp. indica). Science 296, 79–92. doi: 10.1126/science.1068037, PMID: 11935017

[B33] Zhang X. Henriques R. Lin S.-S. Niu Q.-W. Chua N.-H. (2006). Agrobacterium-mediated transformation of *Arabidopsis thaliana* using the floral dip method. Nat. Protoc. 1, 641–646. doi: 10.1038/nprot.2006.97, PMID: 17406292

[B34] Zhang L. Li W. Li Y. Chen B. Wang S. Ma Z. . (2024). Overexpression of *GiLEA5-2.1*, a late embryogenesis abundant gene *LEA3* from *Glycyrrhiza inflata* Bat., enhances the drought and salt stress tolerance of transgenic tobacco (*Nicotiana benthamiana*). Ind. Crop Prod 211, 118308. doi: 10.1016/j.indcrop.2024.118308

[B35] Zhang D. Zhou H. Zhang Y. Zhao Y. Zhang Y. Feng X. . (2025). Diverse roles of MYB transcription factors in plants. J. Integr. Plant Bio 67, 539–562. doi: 10.1111/jipb.13869, PMID: 40013511

[B36] Zhou P. Graether S. P. Hu L. Zhang W. (2023). The role of stress proteins in plants under abiotic stress. Front. Plant Sci. 14. doi: 10.3389/fpls.2023.1193542, PMID: 37215289 PMC10194111

